# Supplementary material for: PINK1-dependent phosphorylation of Serine111 within the SF3 motif of Rab GTPases impairs effector interactions and LRRK2-mediated phosphorylation at Threonine72
Source: Biochem J. 2020 May 11;477(9):1651–68. doi: 10.1042/BCJ20190664 (PMC7219890; doi:10.1042/BCJ20190664)
Supplement: Supplementary Figures S1-S14 [file BCJ-477-1651-s1.pdf]

Supplementary Figure 1. Kinase screen *in vitro* against WT Rab8A:GDP. 100 ng of each protein kinase from the MRC Reagents and Services library was incubated with 2 µg GDP-Rab8A WT or GDP-Rab8A S111A and [γ-^{32}P] ATP for 30 min. Samples were subjected to SDS-PAGE with analysis by Coomassie staining, [γ-^{32}P] incorporation measured by autoradiography with Cerenkov counting and immunoblotting analysis using the indicated antibodies. Proteins highlighted in red are able to phosphorylate GDP-Rab8A at Thr72.

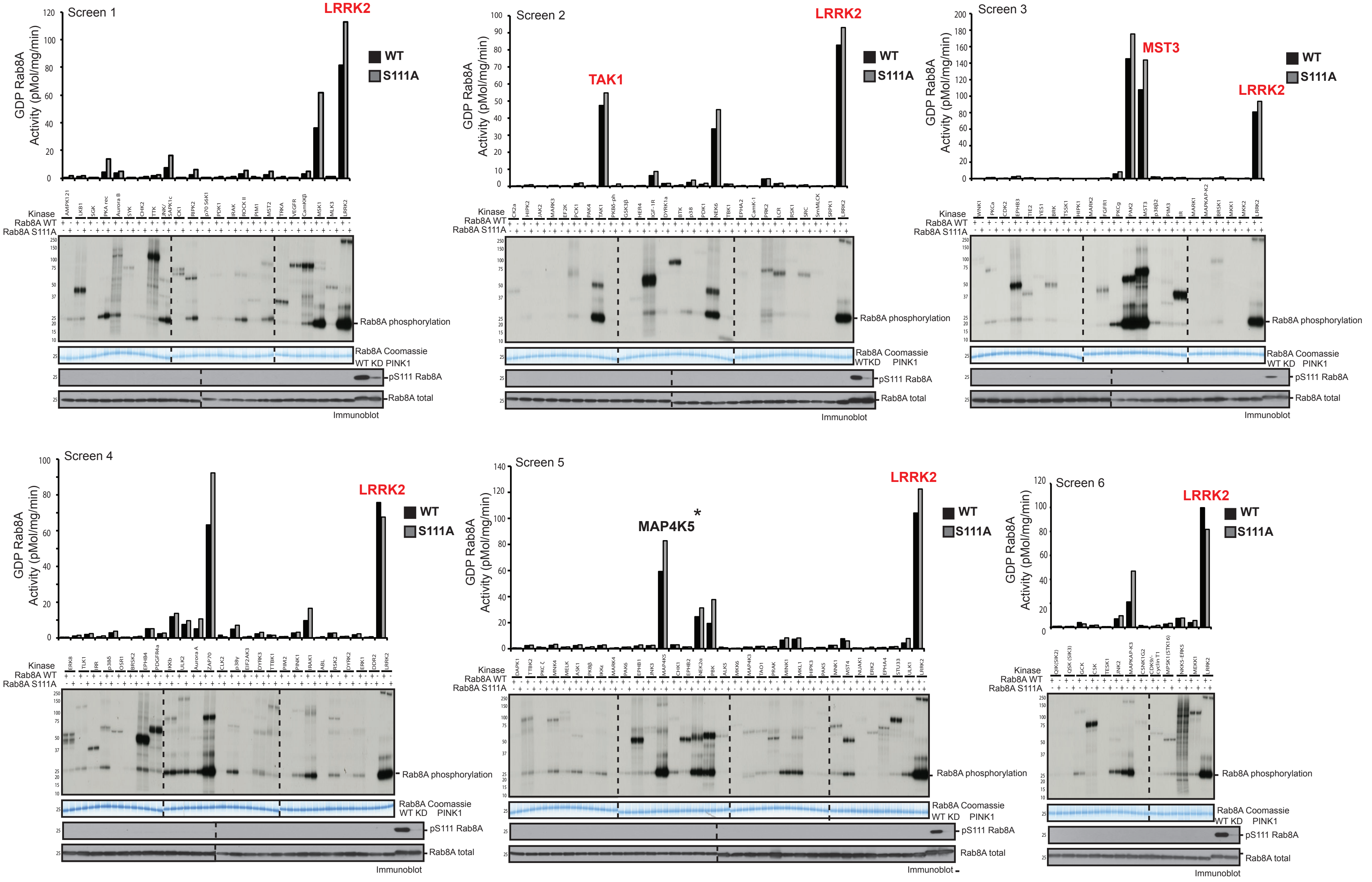

Supplementary Figure 1

Supplementary Figure 2. Kinase screen *in vitro* against WT Rab8A: GppNHp. 100 ng of each protein kinase from the MRC Reagents and Services library was incubated with 2 µg WT Rab8A:GppNHp and [γ-^{32}P] ATP for 30 min. Samples were subjected to SDS-PAGE with analysis by Coomassie staining, [γ-^{32}P] incorporation measured by autoradiography with Cerenkov counting and immunoblotting analysis using the indicated antibodies. Proteins highlighted in red are able to phosphorylate WT Rab8A:GppNHp at Thr72.

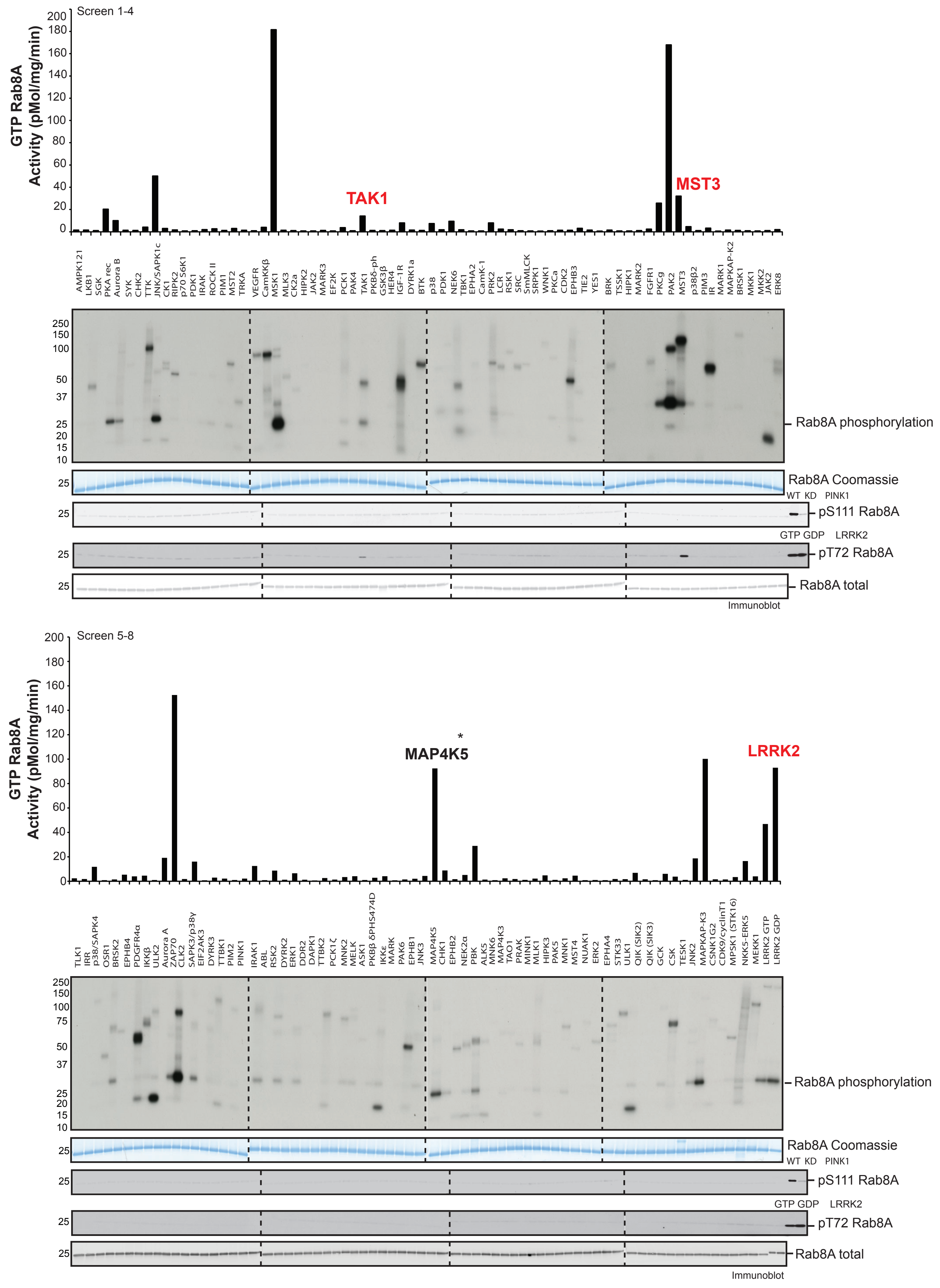

Supplementary Figure 2

Supplementary Figure 3. Structure of Rab8A:GppNHp. Cartoon representation of Rab8A:GppNHp (PDB ID 4LHW) showing the positions of Switch I, Switch II, and RabSF3. Ser111 and Thr72 residues are indicated as spheres. The non-hydrolyzable GTP-analogue GppNHp is shown as a stick model.

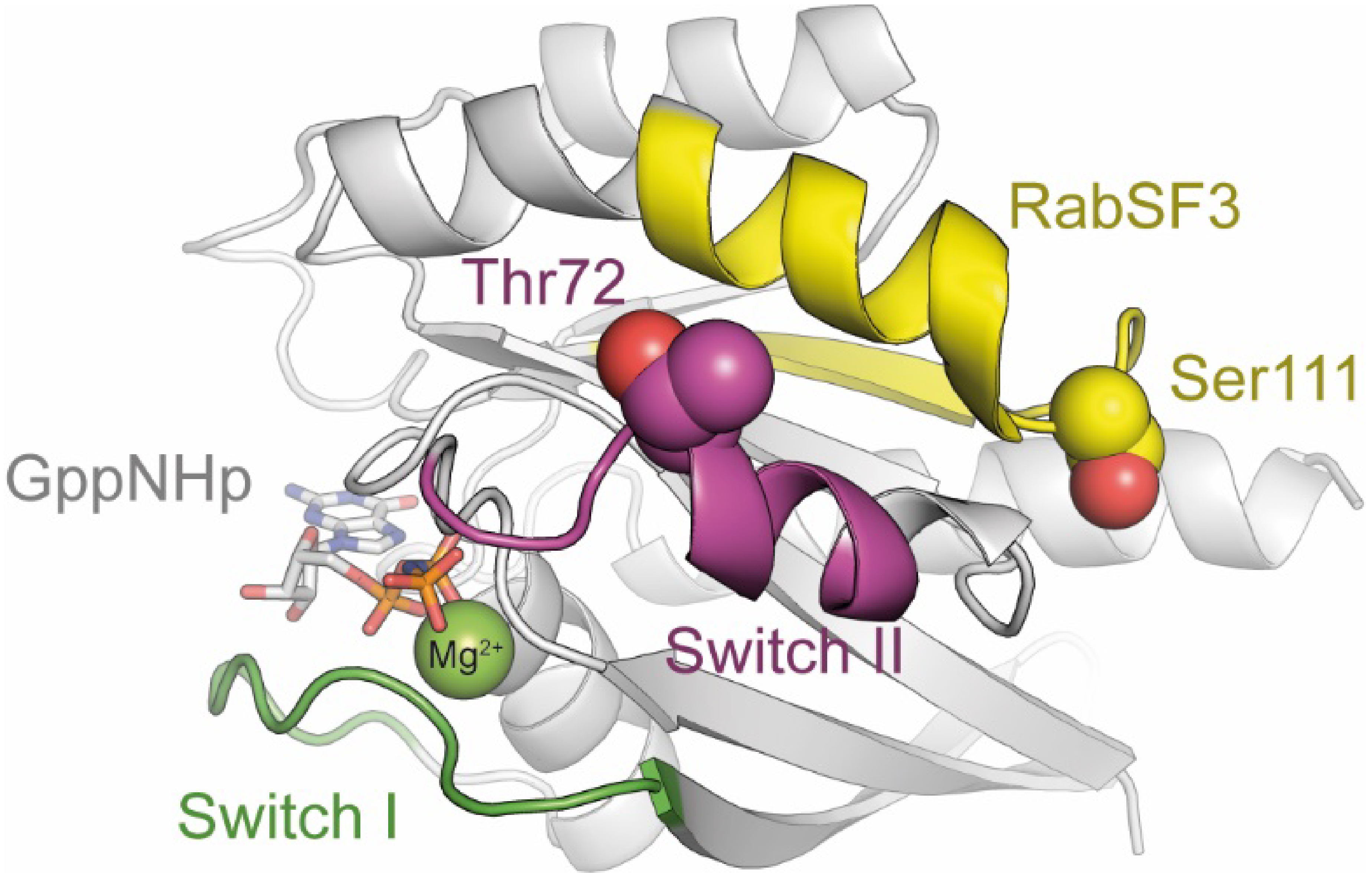

Supplementary Figure 3

Supplementary Figure 4. Expression and purification of Ser111- phosphorylated Rab1B and 8A GTPases. A-B. Analysis of the purity of WT-Rab1B/8a-His_6 and pSer111-Rab1B/8A-His_6 proteins by 12% SDS-PAGE (A). Detection of phosphorylated amino acid residues in WT-Rab1B/8a-His_6 and pSer111-Rab1B/8a-His_6 proteins by Phos-tag™ acrylamide SDS-PAGE. The migration of phosphorylated Rab proteins is retarded (highlighted with red arrows) due to the phos-tag™ compound (B). **C:** Melting points (Tm) of the WT-Rab1B/8a and the pSer111-Rab1B/8a-His_6 proteins determined by a SYPRO® orange-based thermal shift assay. **D:** LC-MS analysis of the WT- and pSer111-Rab proteins used in this study. Note that the N-terminal methionine of Rab1B is usually cleaved off during expression.

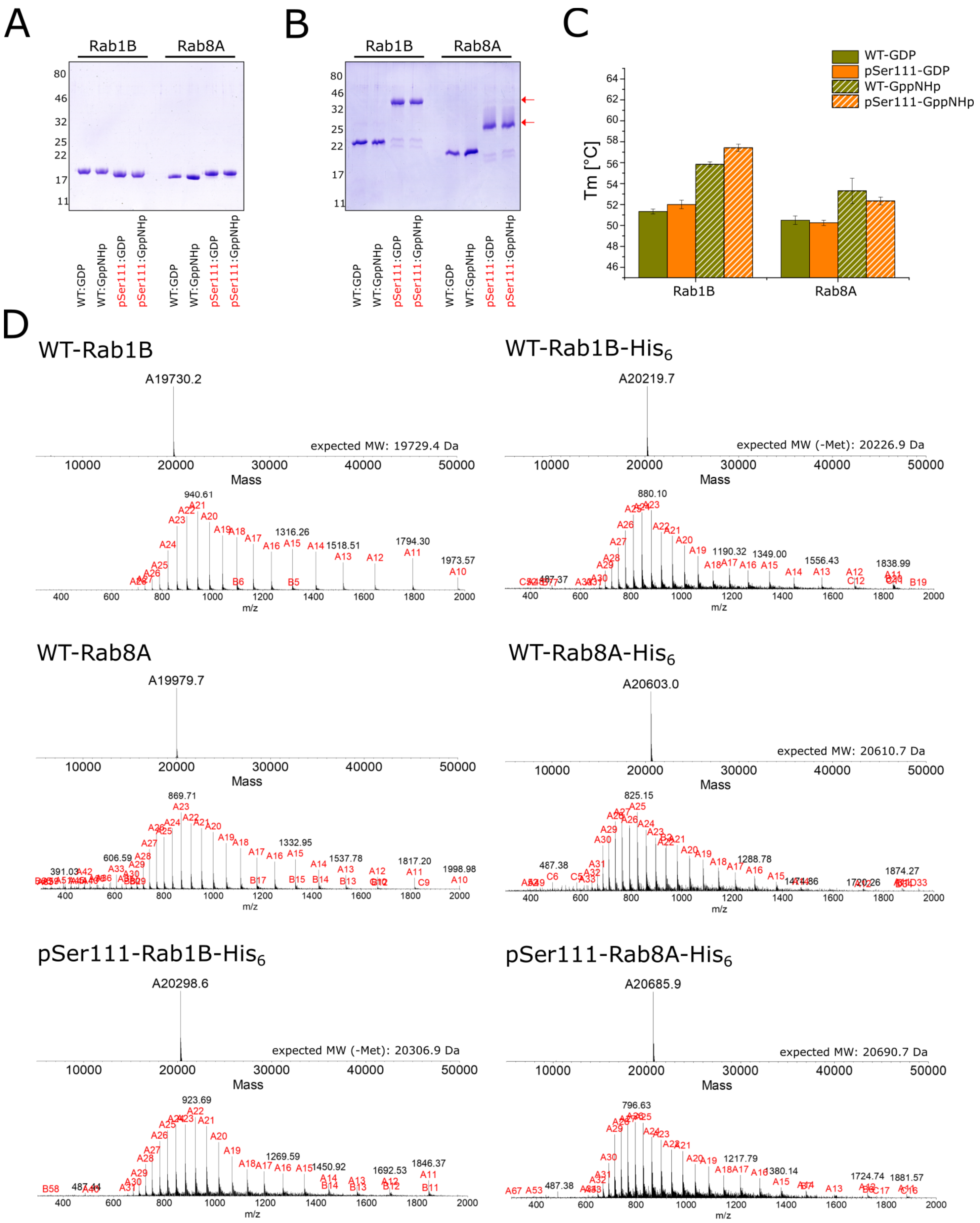

Supplementary Figure 4

Supplementary Figure 5. Rab1B is indirectly phosphorylated by PINK1 at Ser111. A. HEK293 cells stably expressing 3XFlag PINK1 WT were transfected with either Rab8A WT or Rab1B WT and stimulated with 10 µM CCCP for 3 h. 10 mg of whole cell lysate was immunoprecipitated with anti-HA agarose, resolved by SDS-PAGE and stained with colloidal Coomassie. Displayed bands were excised, followed by trypsin digestion and subjected to high performance liquid chromatography with tandem mass spectrometry (LC-MS-MS) on an LQT-Oribitrap mass spectrometer. XIC's display the absolute area (AA) of each Ser111 phosphopeptide of interest, with Y-axis corresponding to phosphopeptide signal intensity and x-axis to the retention time (RT). **B.** HeLa cells were transiently transfected with either HA-Rab1B WT or S111A constructs, followed by 10 µM CCCP or DMSO treatment for 20 h. Samples were subjected to Phos-tag analysis.

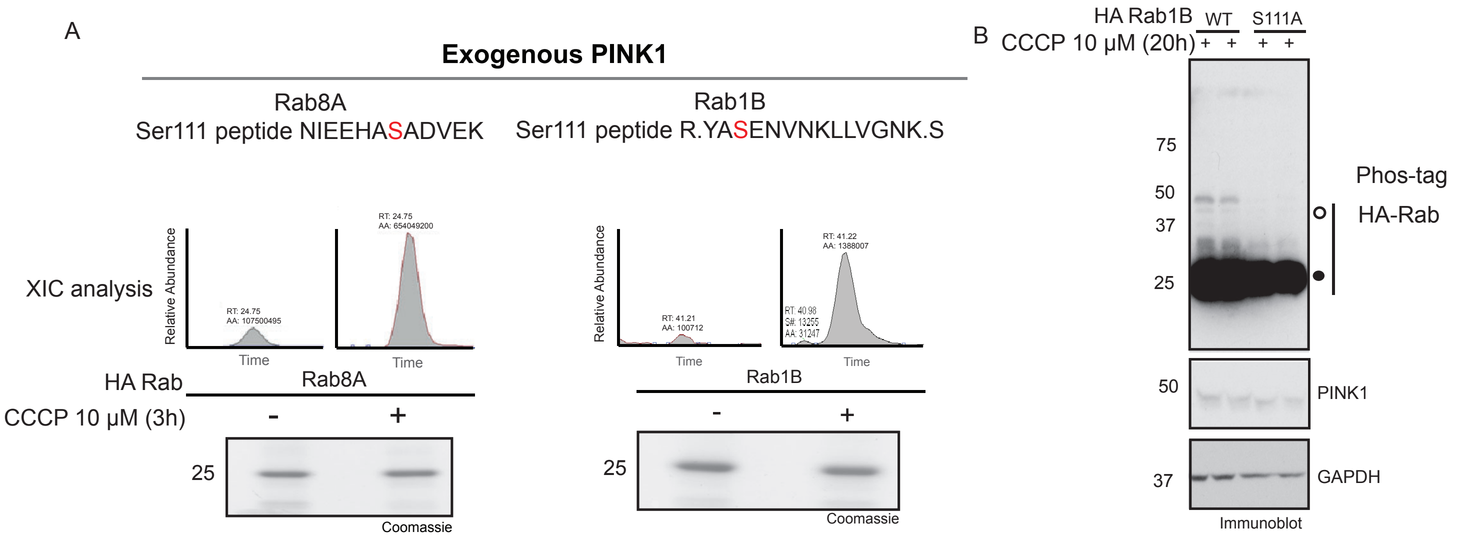

Supplementary Figure 5

Supplementary Figure 6. Rab1B Ser111 phosphorylation impairs GEF and GAP interactions. A, B. (A) The GDP-GTP exchange catalyzed by the GEF DrrA, and (B) the GTP hydrolysis catalyzed by the GAP TBC1D20 (B) were monitored for WT-Rab1B (green) and pSer111-Rab1B-His_6 (teal) proteins via intrinsic tryptophan (Trp) fluorescence. The shown graphs are representative for three independent experiments. C) The nucleotide exchange (GEF activity) and GTP hydrolysis rates (GAP activity) were determined using a single exponential fit, and are represented as % of WT. All data (n=3) are represented as mean ± S.D.

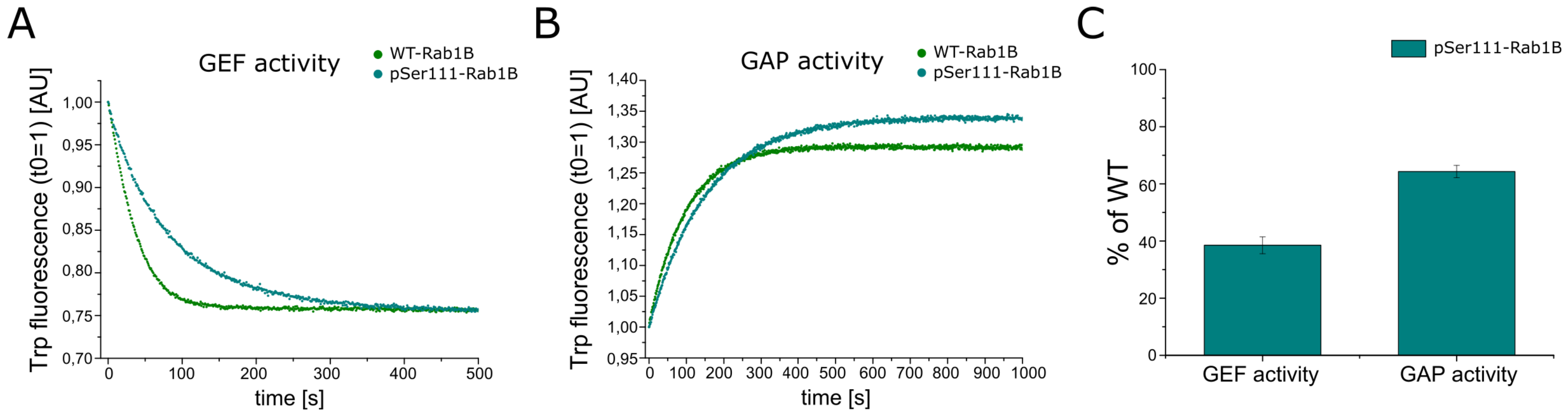

Supplementary Figure 6

Supplementary Figure 7. Binding sites of Rab8A regulators and effectors. A. A depiction of the binding sites of the GEF Rabin8 [31] (teal), the GAP TBC1D20 [44, 45] (purple), and the effector Mical-1 [28] (olive green) on the surface of pSer111-Rab8A:GppNHp (salmon). **B.** Schematic representation of the pSer111-Rab8A amino acid sequence and secondary structure highlighting the binding sites of Rabin8 (teal), TBC1D20 (purple) and Mical-1 (olive green).

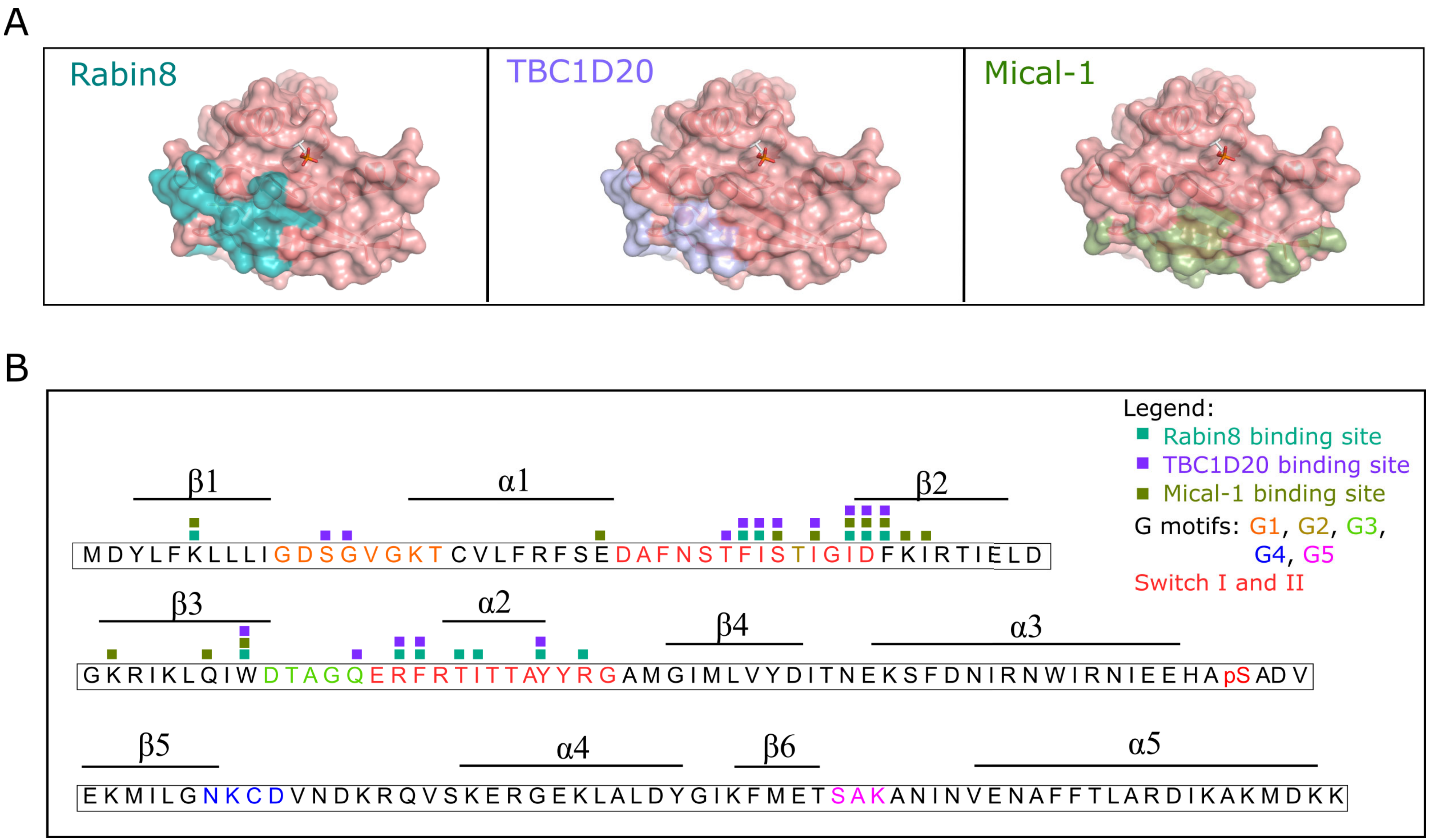

Supplementary Figure 7

Supplementary Figure 8. Rab1B Ser111 phosphorylation disrupts LRRK2-mediated Thr72 phosphorylation *in vitro*. A. 100 ng of recombinant LRRK2 [G2019S] protein was incubated with [γ-^{32}P] and 2 µg of WT or pSer114 Rab1B, in either the GDP or GTP (GppNHp) bound conformation, in the absence or presence of the LRRK2 inhibitor, MLi-2 for 30 min. Samples were subjected to SDS-PAGE, and analysed by either Coomassie staining, [γ-^{32}P] incorporation measured by autoradiography with Cerenkov counting (top panel) and immunoblot analysis, using the indicated antibodies (lower panel). Results are means +/- S.E.M. (*n*=3). **B.** Thr72 specific kinases were assessed for phosphorylation of GDP bound WT or pSer114 Rab1B. 100 ng LRRK2 G2019S, 50 ng TAK1 or 200 ng MST3 were incubated with 2 µg substrate using identical conditions and analysis as in panel A.

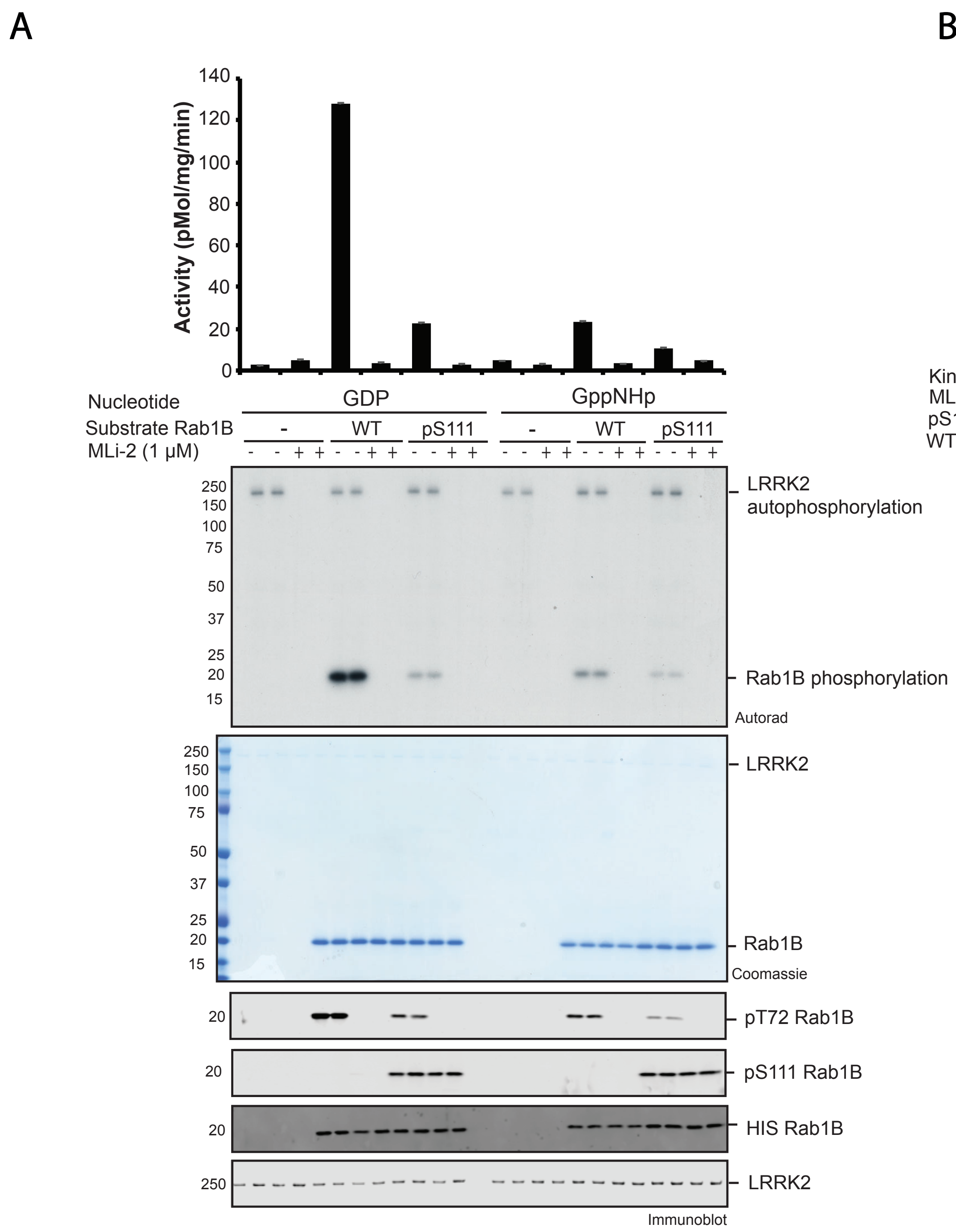

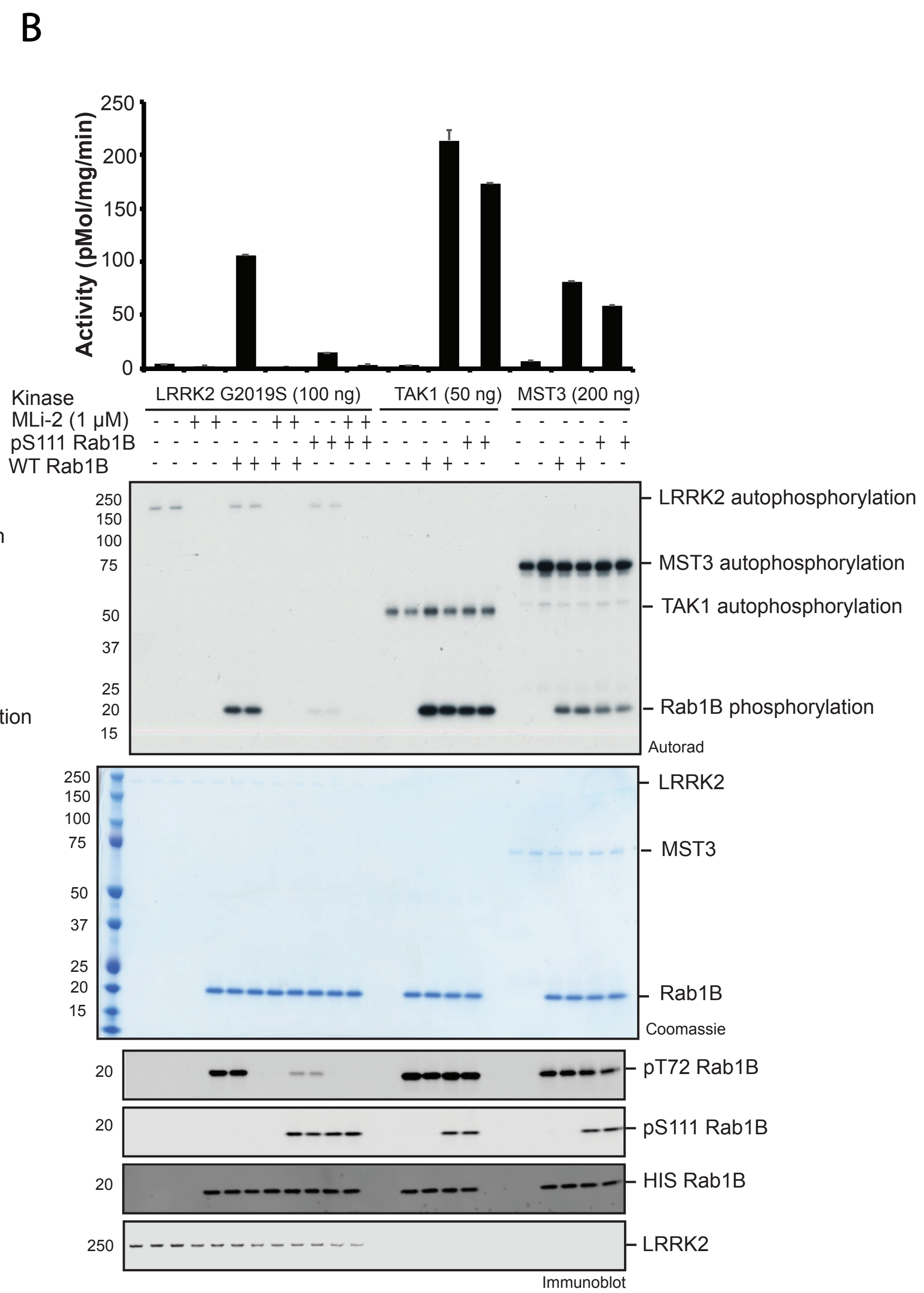

Supplementary Figure 8

Supplementary Figure 9: Superpositions of x-ray structures of Rab8A phosphorylated at Ser111 shown in stereo views. A. Structural superposition of pSer111-Rab8A:GppNHp with pSer111-Rab8A:GDP. An intramolecular salt bridge observed in pSer111-Rab8A:GDP between pSer111 and R79 is indicated by a dotted line. **B.** The nucleotide binding pocket of pSer111-Rab8A:GppNHp. The 2Fo-Fc electron density map of the nucleotide is shown with a mesh representation. **C.** Excerpt of the vicinity of pSer111 in the structure of pSer111-Rab8A:GppNHp. **D.** The nucleotide binding pocket of pSer111-Rab8A:GDP. The 2Fo-Fc electron density map of the nucleotide is shown with a mesh representation. **E.** Excerpt of the vicinity of pSer111 in the structure of pSer111-Rab8A:GDP. An intramolecular salt bridge observed in pSer111-Rab8A:GDP between pSer111 and R79 is indicated by a dotted line.

Supp Fig 9

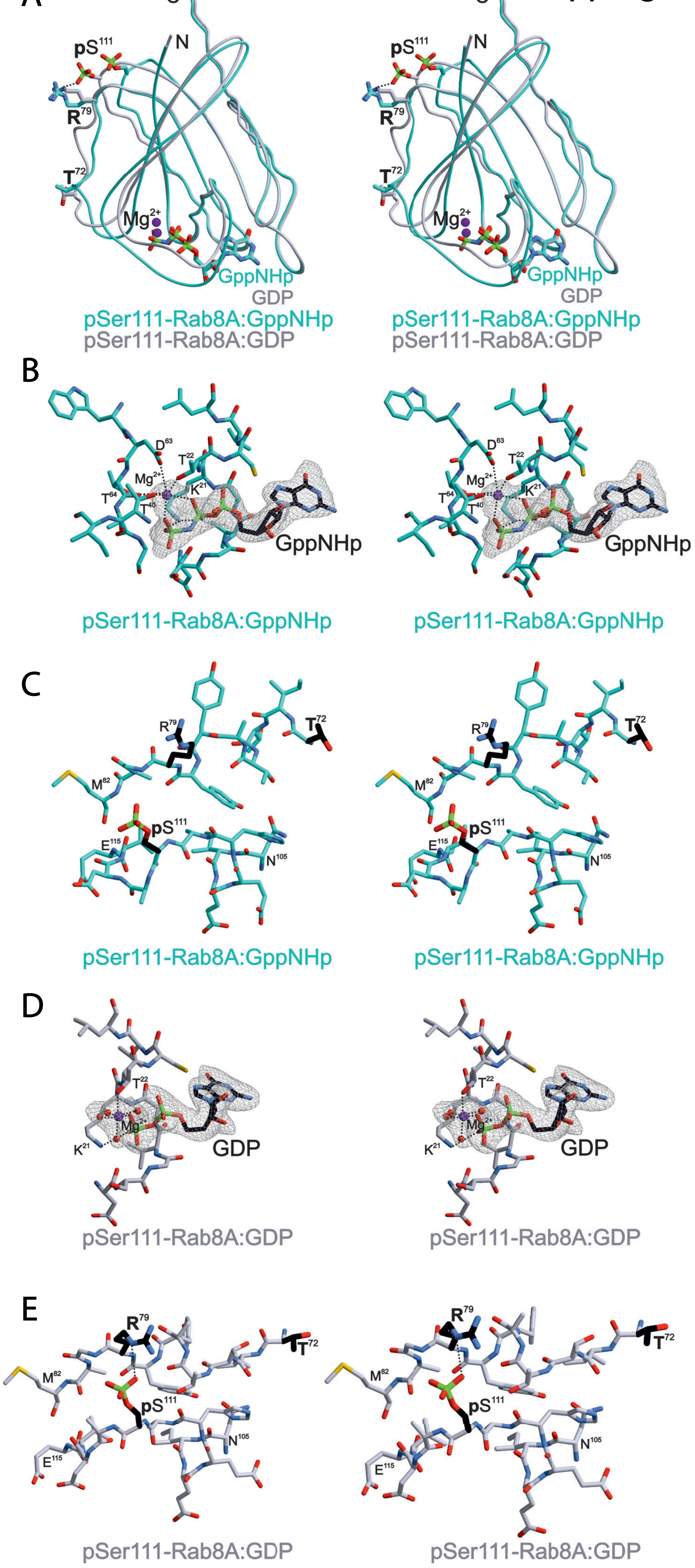

A
C
N
pS111
R79
T72
Mg2+
GppNHp
GDP
pSer111-Rab8A:GppNHp
pSer111-Rab8A:GDP
B
D63
T22
Mg2+
K21
T64
T40
GppNHp
pSer111-Rab8A:GppNHp
C
R79
T72
M82
pS111
E115
N105
pSer111-Rab8A:GppNHp
D
T22
Mg2+
K21
GDP
pSer111-Rab8A:GDP
E
R79
T72
M82
pS111
E115
N105
pSer111-Rab8A:GDP

Supplementary Figure 10. Analytical characterization of ^{15}N-labelled WT- and pSer111-Rab1B proteins. A. Analysis of ^{15}N-labelled WT-Rab1B:GDP/GppNHp proteins by LC-MS and SDS-PAGE (12%). **B.** Analysis of the ^{15}N-labelled pSer111-Rab1B:GDP/GppNHp proteins by LC-MS and SDS-PAGE (12%). Note that the N-terminal methionine of Rab1B is usually cleaved off during expression.

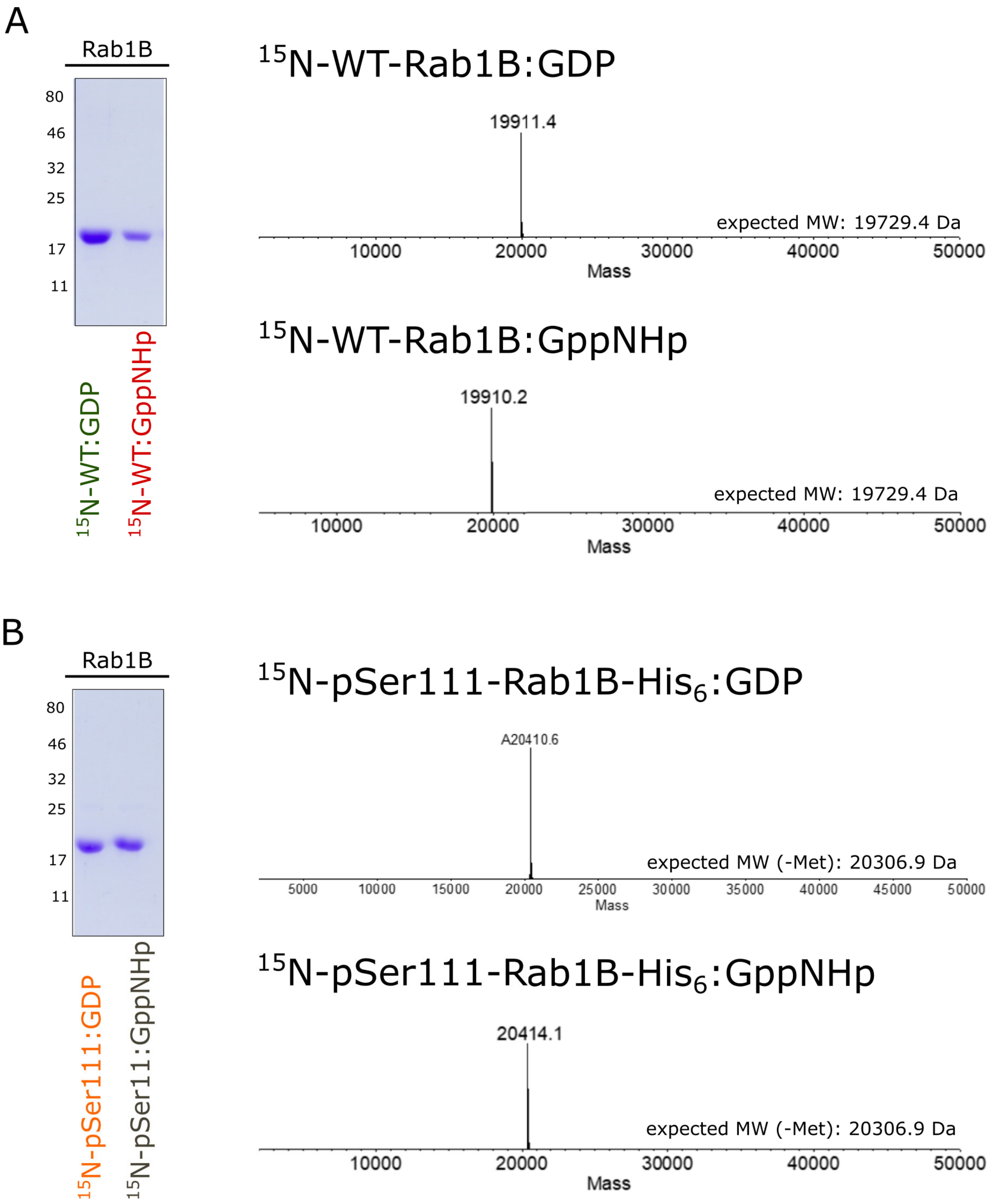

Supplementary Figure 10

Supplementary Figure 11. ^1H-^{15}N HSQC solution NMR spectra of WT- and pSer111-Rab1B spectra in the inactive and active state. Figure insets show the zoom region for central and Trp Hε-Nε. **A.** Overlay spectra of WT-Rab1B:GDP (green) and WT-Rab1B:GppNH (red). Major changes between inactive vs active conformations are shown via overlapping of the WT-Rab1B:GDP and WT-Rab1B:GppNH spectra. **B.** Inactive conformations of unmodified and phosphorylated Rab1B. Overlay spectra shows WT-Rab1B:GDP (green) and pSer111-Rab1B:GDP in orange. Inset shows the central zoom area of the spectra. **C.** Active conformation of unmodified and phosphorylated Rab1B. Overlay spectra of WT-Rab1B:GppNH (red) and pSer111-Rab1B:GppNH (gray).

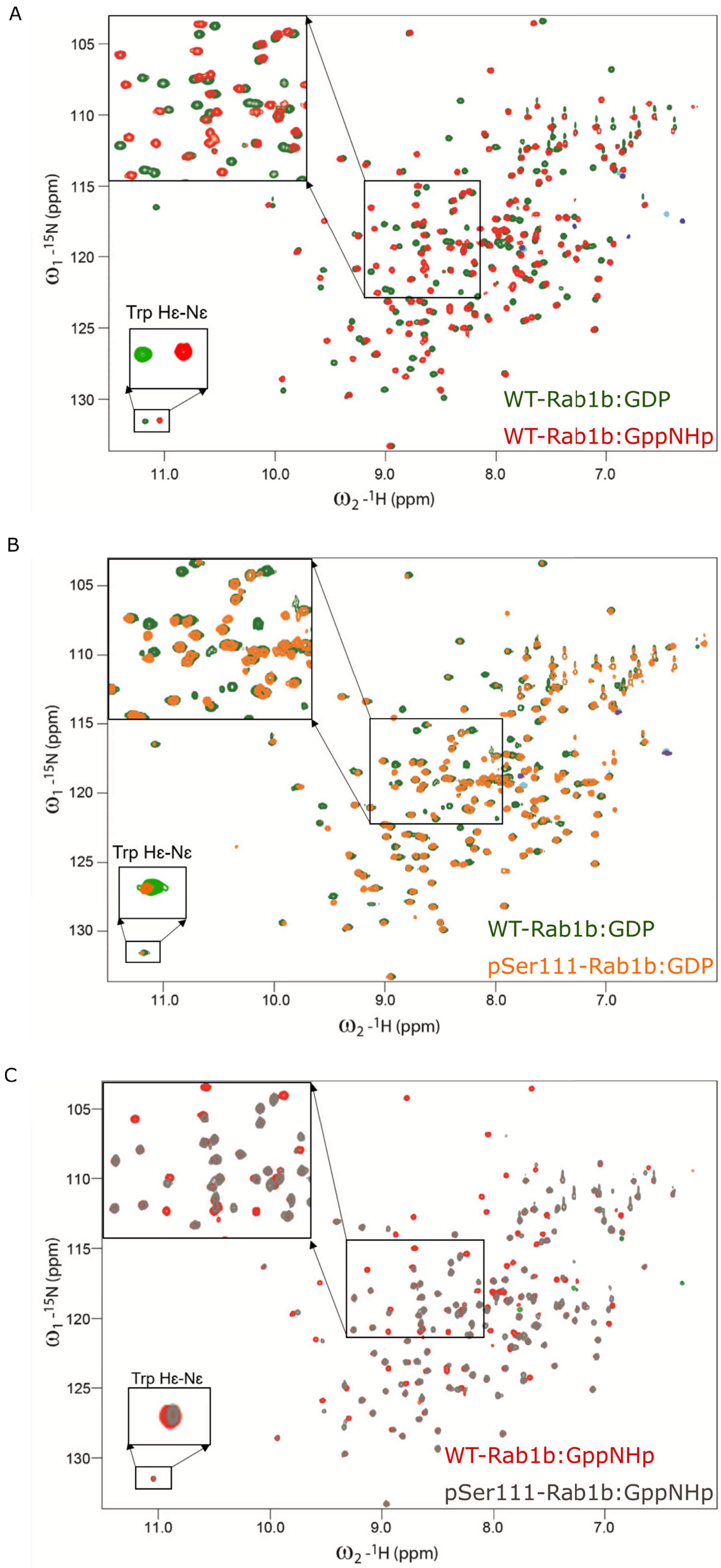

A
105
110
115
120
125
130
Trp Hε-Nε
WT-Rab1b:GDP
WT-Rab1b:GppNHp
11.0
10.0
9.0
8.0
7.0
ω1 -15N (ppm)
ω2 -1H (ppm)
B
Trp Hε-Nε
WT-Rab1b:GDP
pSer111-Rab1b:GDP
C
Trp Hε-Nε
WT-Rab1b:GppNHp
pSer111-Rab1b:GppNHp

Supp Fig 11

Supplementary Figure 12. PINK1 and LRRK2 signalling converge on Rab8A in cells: loading controls and mitochondrial depolarisation readouts. HEK293 Flp-In TREx cells stably expressing PINK1-3XFLAG were co-transfected with 9 µg of LRRK2 R1441G and 3 µg of GFP Rab8A WT or S111A. Cells were treated with DMSO, 100 nM MLi-2 (1.5 h), 10 µM CCCP (3 h) or a combination of each. Cells were lysed and underwent immunoblotting with Phos-tag analysis utilising total antibodies for experimental controls and phospho-specific antibodies as experimental read-outs. Anti-GAPDH and Anti-LRRK2 antibodies confirmed equal loading and LRRK2 expression. Anti-OPA1 antibody confirmed mitochondrial depolarisation across samples. Immunoblots were visualised using the LICOR Odyssey imaging system.

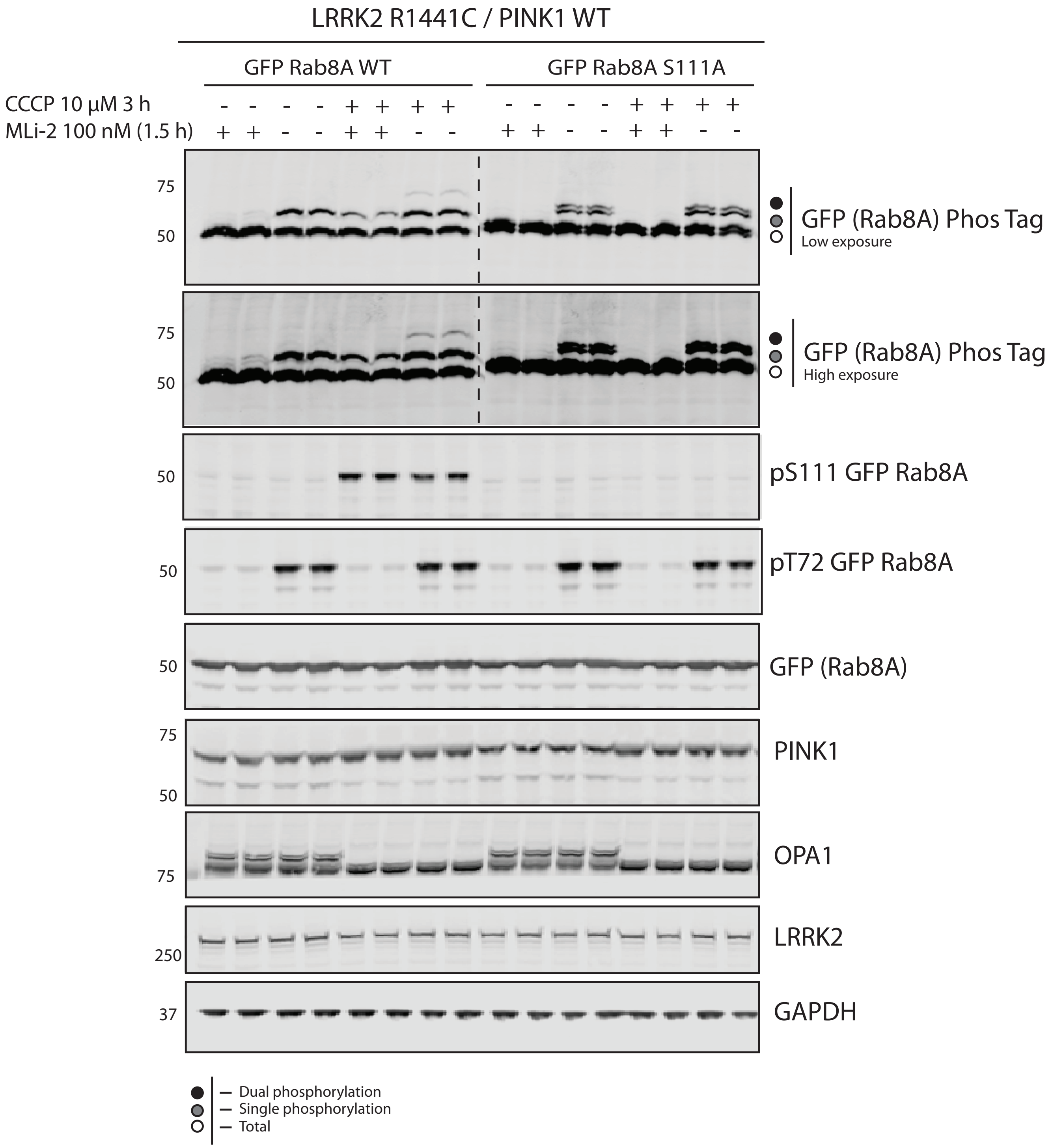

Supplementary Figure 12

Supplementary Figure 13. PINK1 and LRRK2 signalling converge on Rab8A in cells: quantification. Secondary antibodies conjugated with LI-COR IRDye enabled quantification with LICOR Odyssey software. **A.** Percent of mono-phosphorylated Rab8A. **B.** Percent of dual phosphorylated Rab8A of total Rab8A signal. **C.** Percent of dual phosphorylated Rab8A versus predicted.

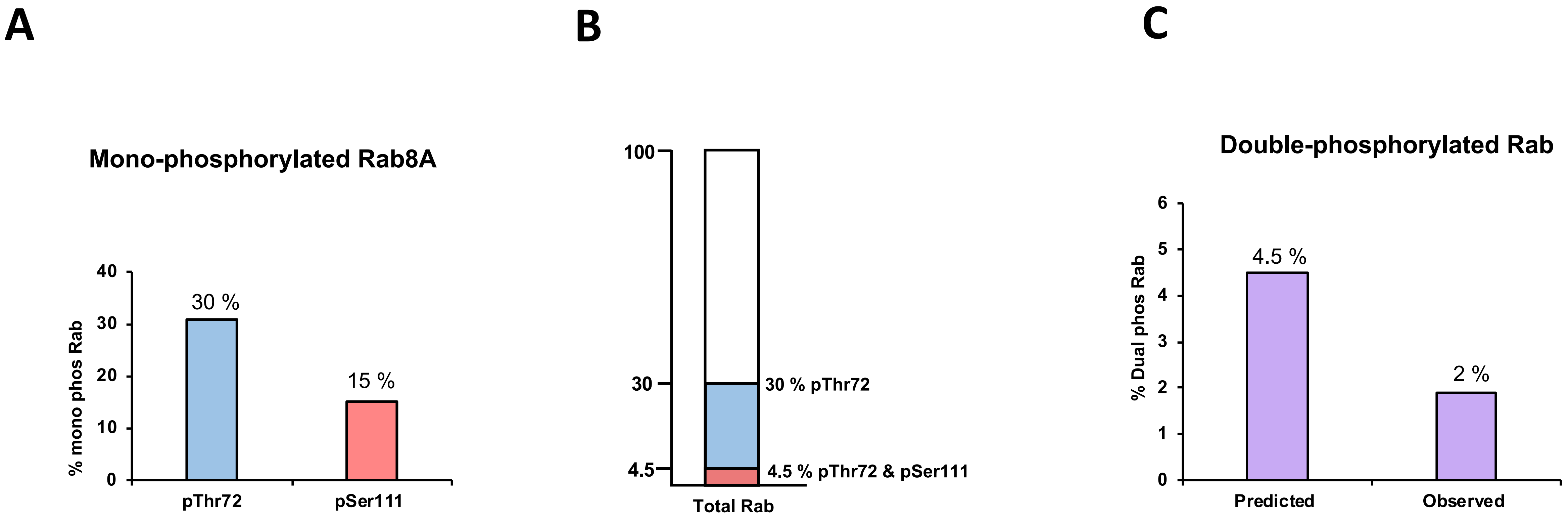

Supplementary Figure 13

Supplementary Figure 14. The PINK1 catalytic-inactive [D384A] mutant abolishes Ser111 Rab8A phoshphorylation and LRRK2 signalling converge on Rab8A in cells. HEK293 Flp-In TREx cells stably expressing wild-type or D384A kinase-inactive PINK1-3XFLAG were co-transfected with 9 µg of LRRK2 R1441G and 3 µg of GFP Rab8A WT or S111A. Cells were treated with DMSO, 100 nM MLi-2 (1.5 h), 10 µM CCCP (3 h) or a combination of each. Cells were lysed and underwent Phos-tag analysis with total and phospho-specific antibodies using the LICOR Odyssey imaging system for detection. IRDye 800CW fluorescent secondary antibody was utilised for GFP Rab total signal, whilst IRDye 680RW fluorescent antibody was used for the specific phospho-Rab detection. Presence of yellow fluorescence upon multiplexing with Image Studio software, indicates the presence of site-specific phosphorylated Rab within the GFP total Rab population.

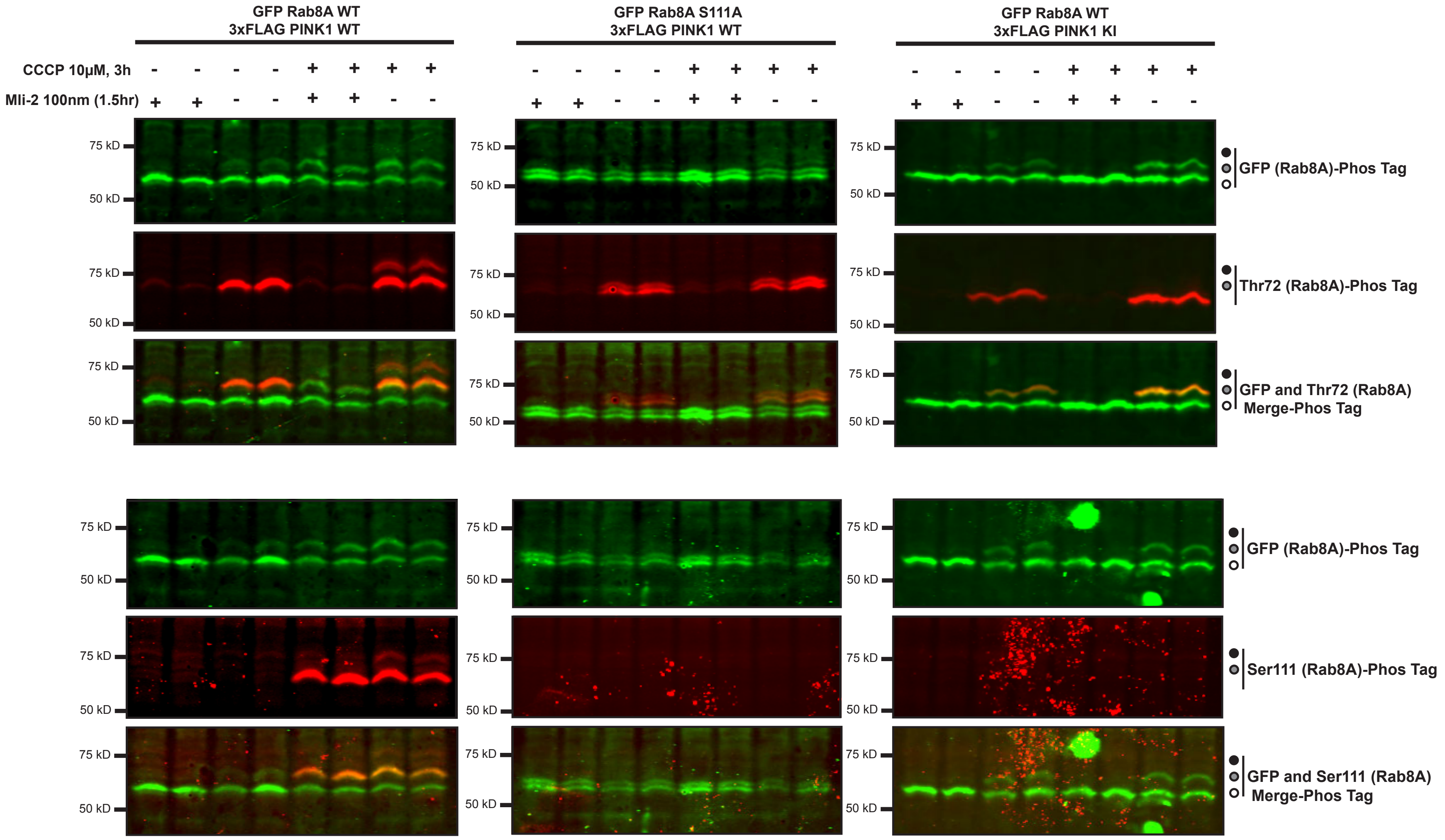

Supplementary Figure 14
